# Production of Uniform Droplets and Lipid Nanoparticles Using Perfluoropolyether-Based Microfluidic Devices

**DOI:** 10.3390/mi16020179

**Published:** 2025-01-31

**Authors:** Mincheol Cho, Eun Seo Kim, Tae-Kyung Ryu, Inseong Choi, Sung-Wook Choi

**Affiliations:** 1 Biomedical and Chemical Engineering, The Catholic University of Korea, 43 Jibong-ro Wonmi-gu, Bucheon-si 14662, Gyeonggi-do, Republic of Korea; 2 Department of Neurology, Institute for Cell Engineering, Johns Hopkins University School of Medicine, Baltimore, MD 21205, USA

**Keywords:** perfluoropolyether, microfluidics, lipid nanoparticles, droplet

## Abstract

Microfluidic devices are greatly affected by the materials used. The materials used in previous studies had problems in various aspects, such as processing, adsorption, and price. This study will investigate the materials needed to overcome such problems. Various microfluidic devices based on the perfluorinated compound perfluoropolyether (PFPE) were fabricated and mixed with hydrophilic and amphiphilic monomers, including poly(ethylene glycol) diacrylate, polyethylene glycol monomethacrylate, poly(ethylene glycol) methyl ether methacrylate, acrylic acid, and 2-hydroxyethyl methacrylate. A PFPE-based sheet with a repeating structure of hydrophobic and hydrophilic groups was fabricated. Thus, the hydrophilicity of highly hydrophobic PFPE was enhanced. The fluidic channel was engraved on a PFPE-based sheet using laser cutting and a fabricated microfluidic device. The channels of microfluidic devices are micro-scale (100 µm~300 µm). The lipid nanoparticles and droplets generated through the microfluidic device demonstrated uniform particles continuously.

## 1. Introduction

Over the past decade, microfluidic technology has advanced in various directions (droplet generation, organ-on-chip, hydrogel chip, drug screening, and detection equipment) [1,2,3,4]. Microfluidic technology offers several advantages compared to laboratory-scale analysis. In microfluidic systems, fluids are processed at the micrometer level, with total flow rates in microliters per minute. Because of this characteristic, there are benefits such as minimizing reagent consumption, reducing manufacturing costs, and a larger volume-to-surface area ratio, which leads to higher reaction efficiency and shorter reaction times [5]. Additionally, the precise control of flow rates allows for high reproducibility and reliability in various applications using microfluidic chips. Microfluidic chips are powerful tools for creating uniform and small-sized LNPs (lipid nanoparticles) and emulsions [6]. Due to these advantages, there has been a surge in research using microfluidic devices to produce uniform LNPs and emulsions.

The structure and materials used in microfluidic chips are important research areas for advancing device applications. Materials have a significant impact on microfluidic technology, leading to the development of various types of materials. Commonly used materials in current research include PDMS (polydimethylsiloxane), PMMA (polymethyl methacrylate), silicon, and glass [7,8]. In fluidic devices, the primary material used for laser engraving is PMMA. However, PMMA has poor solvent resistance and forms bumps during laser cutting [9]. The microchannels of the polydimethylsiloxane microfluidic device had a blackened and rough surface after laser engraving [10]. Glass is considered the most advantageous material for microfluidic devices due to its transparency and physicochemical properties. However, it has the drawback of rough channel surfaces, and is difficult to handle due to the high pressure required during channel fabrication [11]. Organic materials like PDMS and PMMA do not exhibit resistance to organic solvents (acetone, benzene, and alcohol) [12]. In particular, PDMS is attributed to numerous outstanding characteristics, including biocompatibility, elastomeric properties, gas permeability, optical transparency, the ease of molding and bonding with glass, and low manufacturing costs [13,14]. However, due to the hydrophobicity of PDMS, it is difficult to inject aqueous solutions into the microchannels of PDMS-based devices. Hydrophobic substances can easily absorb onto the PDMS surface, interfering with analysis [15]. Consequently, surface modification is important for successfully suppressing the nonspecific adsorption of hydrophobic species and improving wettability. To address these drawbacks, research is also being conducted on hybrid materials, such as silicone and glass, silicone and polymers, or glass and polymers, for the fabrication of microfluidic devices [3].

Wu and Hjort’s research team studied surface modification by adding surfactants to the PDMS prepolymer before curing, improving the surface hydrophobicity of PDMS and preventing the nonspecific adsorption of biomolecules [16]. Jonker et al. tested the hydrofluoric acid (HF) etching of powder-blasted (abrasive jet machined) in glass microfluidic chips to prevent cracking due to high pressure. Despite these studies, challenges remain, such as microfluidic channel clogging, the sensitivity of polymer materials to temperature, and the complexity and high cost of manufacturing processes for multilayer microstructures or complex microfluidic devices [11].

To overcome these limitations, fluorinated elastomers such as PFPE (perfluoropolyether) can be considered alternative materials for microfluidic devices. PFPE possesses several attractive characteristics, including low surface energy, high elasticity, high gas permeability, and biocompatibility [17,18]. Due to its low surface energy and chemical resistance, it can prevent molecular adsorption and is being studied as an anti-fouling material. Furthermore, the oxygen in the backbone of PFPE polymers provides molecular flexibility and a low melting point [19,20]. Additionally, during laser cutting, when engraving the microchannel, there is no bump on the surface caused by heat. As a result, the manufacturing process for PFPE-based formulations allows the PFPE to exist in a liquid state at room temperature, making it possible to produce them in various forms. In this study, the physical surface properties of microfluidic chips were controlled by utilizing various hydrophilic monomers based on PFPE. Microfluidic patterns were fabricated using laser engraving, and the applicability of the hydrophilically modified microfluidic chips was confirmed for LNPs and uniform emulsions (O/W). Through the various structures and surface properties of PFPE-based microfluidic chips, depending on the monomers mixed, they could be used not only for the production of LNPs or uniform emulsions but also for chemical and biomolecular synthesis [21].

## 2. Materials and Methods

### 2.1. Materials

PDMS elastomers and curing agents (Sylgard^®^ 184) were purchased from Sewang Hitech (Gimpo, Republic of Korea). PFPE (Fluorolink MD 700, Solvay, Brussels, Belgium), the PDMS elastomer and curing agent (Sylgard^®^ 184) were purchased from Sewang Hitech (Gimpo, Republic of Korea); acrylic acid (AA, anhydrous, contains 200 ppm MEHQ as inhibitor, 99%, Sigma-Aldrich, St. Louis, MO, USA), poly(ethylene glycol) diacrylate (PEGDA, Mn = 700, Sigma-Aldrich, St. Louis, USA), poly(ethylene glycol) methyl ether methacrylate (PEGMA, Mn = 950, Sigma-Aldrich, St. Louis, USA), 2-hydroxyethyl methacrylate (HEMA, TCI, Tokyo, Japan), polyethylene Glycol Monomethacrylate (mPEG, TCI, Tokyo, Japan), and 2-hydroxy-2-methylpropiophenone (Darocur 1173, Sigma-Aldrich, St. Louis, USA) were used to fabricate the microfluidic device. We used Kolliphor^®^ P 188 (solid, Sigma-Aldrich) and isododecane (mixture of isomers, tech. 80%, Alfa-Aesar, Ward Hill, MA, USA) to fabricate the emulsion and BupH™ phosphate-buffered saline (PBS, Thermo Fisher Scientific, Waltham, MA, USA, pH 7.2), EtOH, 1,2-Dipalmitoyl-sn-glycero-3-phosphocholine (DPPC, Avanti Polar Lipids, Inc., Alabaster, AL, USA), cholesterol (Avanti Polar Lipids, Inc.), and Dulbecco’s phosphate-buffered saline (DPBS) were used to fabricate the LNPs.

### 2.2. Fabrication of PDMS, PFPE, PFPE Co-Polymer Slab

A PDMS mixture (PDMS elastomers and curing agents in a 10:1 weight ratio) was poured into a Petri dish. After degassing for 30 min, the Petri dish was placed in an oven at a temperature of 60 °C for 5 h for curing, after which a PDMS slab was obtained. The 0.5 T silicon sheet was cut L-shaped and placed between 3T glass plates to make an aperture (9 mm × 9 mm) for injecting the PFPE-based mixture and fixed using double clips. The PFPE-based material comprises six types: plain PFPE, PEG-DA, PEG-MA, mPEG, AA, and HEMA (5, 10, 15, and 20 wt% against PFPE), and the photoinitiator was thoroughly mixed with a spatula (a mixture of PFPE and mPEG was double boiled in 60 °C water). After degassing, the mixture of PFPE containing the polymer or monomer was injected into the aperture of two glass plates, followed by UV exposure (Inno cure 850) for 70 s. The cured slab was peeled off from the glass plates and washed with ethanol. The obtained PDMS and PFPE-based material slabs were engraved using the CO2 laser (40 W, ML4040, Machine Shop, Paju-si, Republic of Korea). The laser power ranged from 6.6 to 7.3% at a speed of 1.0 mm/s and ranged from 7.6 to 8.1% at a speed of 5.0 mm/s.

### 2.3. Fabrication of Microfluidic Device

The microfluidic device consists of one acrylic board, one slide glass layer, two PDMS layers, and two PFPE-based layers. A glass slide was used as the bottom of the fluidic device to press harder and observe the junction and channel. The second layer, a typical 1T PDMS slab without any pattern, was utilized to uniformly distribute the pressure applied to the microfluidic device. The third layer, which is a PFPE-based material slab, had microchannels engraved using the CO_2_ laser (depth; 200 μm, width; 100~150 μm). On top of this, a PFPE-based slab with inlet and outlet holes was placed to cover the microchannel. For the fifth layer, we used 4 T PDMS to tube the inlet and outlet. A 3 T transparent PMMA slab was used for the top layer of the microfluidic device, which can press hard and observe the microfluidic device. Due to the limitations of creating small holes in the glass with a CO_2_ laser, PMMA, which had inlet and outlet holes, was used.

### 2.4. Characterization of PFPE-Based Slabs

The contact angle was measured on static drops of water on different substrates using ImageJ 1.54m (National Institutes of Health, Bethesda, MD, USA). The weight and length swelling ratio were measured from the difference in weight and length before and after the immersion of the PDMS or PFPE-based material slabs (10 mm × 10 mm). The transparency of the PFPE-based material slab was captured by optical microscopy (BX-43, Olympus, Tokyo, Japan).

### 2.5. Production of Emulsion Droplets and Lipid Nanoparticles

For the oil-in-water emulsion, isododecane and poloxamer (3 wt %, Kolliphor^®^ P 188) were used for the discontinuous and continuous phases, respectively. The effect of the production speed (TFR; 5.5–10 mL/h) and the organic-to-aqueous ratio (FRR; 1:10 to 1:20) was evaluated.

The emulsion droplet formation was observed using a high-speed camera (CR3000×2, Optronis, Kehl, Germany) to obtain time-lapse images. The produced droplets were collected on a concave glass and observed through optical microscopy. The average sizes and standard deviations of the droplets were calculated using ImageJ 1.54m (*n* = 100). The coefficient of variation (CV) was calculated by dividing the standard deviation by the average value.

For LNPs, DPPC and cholesterol were used for the discontinuous phase, and PBS was used for the continuous phase. LNPs were prepared using a microfluidic device, where the total flow rates (TFR; 2 to 10 mL/h) and organic-to-aqueous ratio (FRR; 1:3) were evaluated.

The size of LNPs, PDI, LNPs’ size distribution, and zeta potential were measured using Malvern Zetasizer nano ZS (Malvern Instruments Ltd., Worcestershire, UK) and the DLS technique.

The inlet/outlet channels were connected to a syringe pump (KDS-100-CE, Harvard Bioscience Inc., Holliston, MA, USA). The precipitates in the channel were observed using a high-speed camera to obtain time-lapse images.

## 3. Results and Discussion

### 3.1. Fabrication of the Microfluidic Device

**Figure 1:**
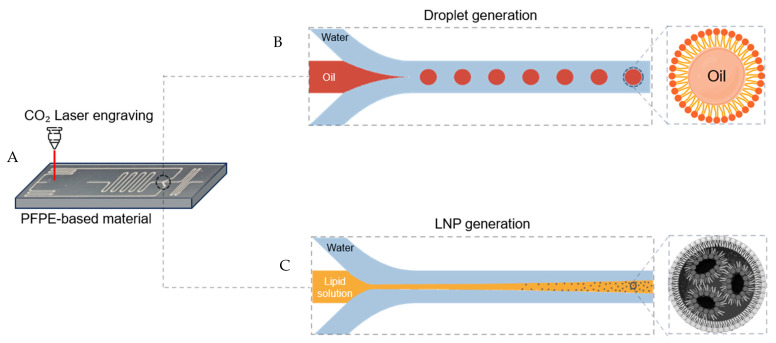
Schematic illustration of (**A**) fabrication, (**B**) emulsion, and (**C**) the production of LNPs in a microfluidic device.

Figure 1A provides schematic illustrations of the laser engraving of the PFPE-based material slab. By utilizing a laser, precise patterns can be engraved quickly and efficiently, offering the advantages of cost-effectiveness and accessibility. To reduce the back pressure of the microfluidic device channel, the junction was engraved with a narrow, small volume, and the channel was printed broad with a large volume. Describing the structure of the flow, there were two inlets for the introduction of continuous and discontinuous phases and an outlet for the outflow of the resulting LNPs and emulsion droplets. The continuous phase flowed in an inlet hole and was divided into two flow streams. The continuous phase was in contact with the discontinuous phase at the Y-junction and formed emulsion droplets or LNPs. Fabricating LNPs just by diffusion would take days, so we improved the mixing efficiency through the winding channel [22]. There was no leak in the liquid without other glue. Figure 1B,C show uniform droplet generation and LNPs being fabricated using the microfluidic device.

### 3.2. Characteristics of the PFPE-Based Material Slab

Generally, hydrophilic and hydrophobic materials are known as proper components for the O/W emulsion and W/O emulsion systems, respectively [23]. There has been research on hydrophilic modification methods for PDMS, which can be broadly categorized into three main approaches. The first is gas-phase treatment, including plasma treatment, UV treatment, and chemical vapor deposition (CVD) [24,25,26]. Valessa Barbier’s research team activated the surface with Ar plasma pretreatment, followed by acrylic acid (AAc) plasma treatment to coat the surface with acrylic acid. The coated polymer film was then cross-linked to enhance cohesion, limiting the recovery of hydrophobicity. Unfortunately, after being stored in the air for three days, the hydrophobicity returned to the original PDMS level [24]. The second approach involves wet chemical methods, including the LBL (layer-by-layer) deposition, sol–gel coating, silanization, dynamic surface modification, and deliberate protein adsorption [27,28,29]. In the research by G. Mehta’s team, the LBL method was used to modify the surface of PDMS with polyelectrolyte multilayers (PEMs). However, the stability of PEMs can vary depending on environmental factors, such as temperature, pH, ionic strength, and the solution concentration [27]. The final approach is a combination of gas-phase and wet chemical methods to improve hydrophilicity. These studies also have limitations, such as a lack of versatility, complex processes, or the recovery of hydrophobicity. Consequently, to address these disadvantages, we conducted research using PFPE as a substitute for PDMS. By adding a carboxyl-functionalized additive to PFPE and curing it, we imparted hydrophilicity to the PFPE.

**Figure 2:**
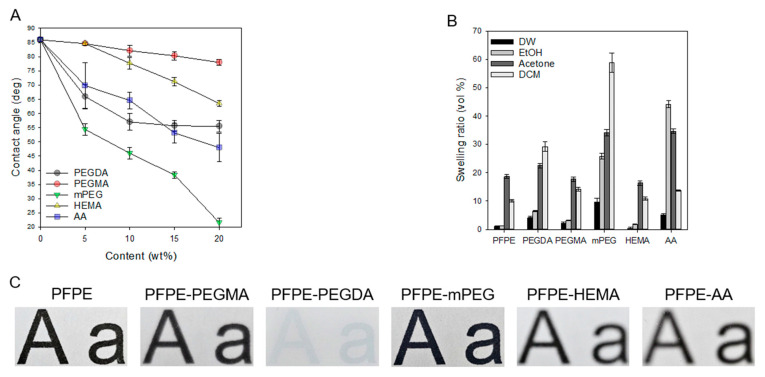
(**A**) Water contact angles, (**B**) swelling ratio, and (**C**) photographs of PFPE-based slabs with different contents of PFPE-based materials (*n* = 5).

Contact angles for PFPE-based materials were measured in proportion to the amount of additive. The hydrophilicity of PFPE-based material slabs increased following the content of the additive (Figure 2A). The swelling property was characterized by immersing PFPE-based material slabs in various solvents (Figure 2B).

If the microfluidic chip has a long usage time with the solvent, and if the slab has been swelling, the channel is narrowed, and the condition of the chip inside can be changed. For the solvent of the swelling test, DW was used for CP; EtOH and DCM were used for DP; and acetone was measured because of the general solvent.

The transparency of each PFPE-based material slab (20 wt%) was captured from the 2 mm gap between the PFPE-based material slab and the text (Figure 2C). A transparency photo was posted to select the observable material by optical microscopy.

Evaluating the suitability of the material in microfluidic devices using the contact angle and swelling ratio, PFPE-HEMA was found to be adequate for the fabrication of LNPs, and PFPE-AA was adequate for droplet generation. We measured the swelling ratio of the mPEG-PFPE slab, and the lowest water contact angle was higher than other PFPE-based material slabs for each solvent. The high swelling ratio can strain the channel into a narrow channel, and back pressure can occur. Furthermore, the mPEG-PFPE slab was destroyed after the swelling test in DCM. Thus, mPEG-PFPE is not suitable for microfluidic devices. PFPE-PEGMA was not used for microfluidic devices because the contact angle showed the highest degree. PFPE-PEGMA showed the lowest degree of improvement in hydrophilicity. Since PFPE-HEMA in ethanol had the lowest swelling ratio and PFPE-AA in DCM had the second lowest swelling ratio, but the hydrophilicity greatly improved compared to PFPE-HEMA, PFPE-HEMA was used for LNPs, and PFPE-AA was used for the emulsion.

### 3.3. Synthesis of Lipid Nanoparticles

**Figure 3:**
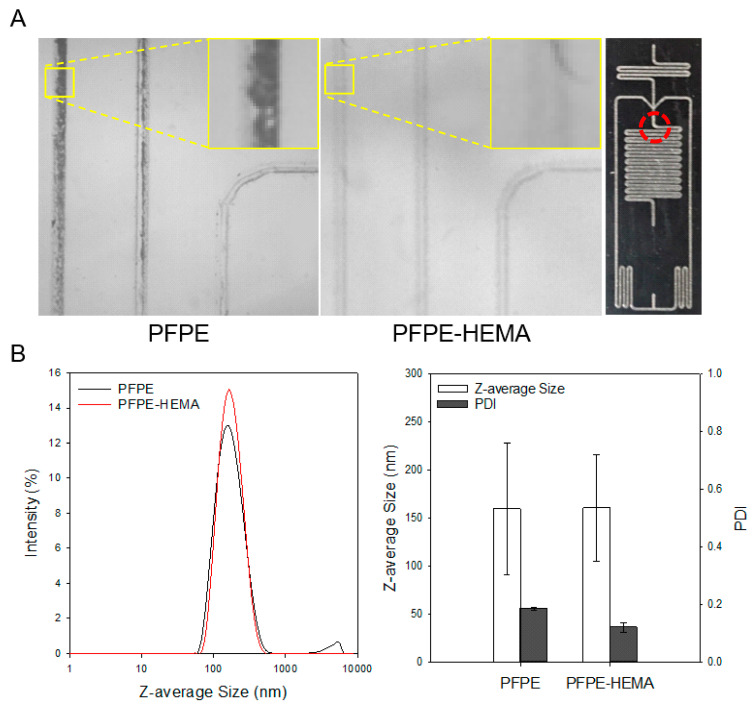
(**A**) Snapshot of PFPE-based slab channel, where fabricating LNPs were captured by high-speed camera and slab design for fabricating LNPs. (**B**) Z-average size and PDI of LNPs obtained by different PFPE-based material slabs.

Figure 3 shows a comparison image of optical microscopy and the Z-average size of LNPs and PDI depending on PFPE and PFPE-HEMA. LNPs were fabricated at production speed (TFR; 2 mL/h), and the organic-to-aqueous ratio (FRR; 1:3) using PFPE, the PFPEP-HEMA slab, and each channel was compared by optical microscopy. The PFPE-HEMA slab had a clean channel wall, but the PFPE slab had precipitates on the channel wall (Figure 3A). The mean Z-average size and PDI of LNPs were fabricated at the production speed (TFR; 10.0 mL/h) and the organic-to-aqueous ratio (FRR 1:3) were measured by DLS. The average size of nanoparticles is similar for both PFPE-based materials (PFPE; 159.6 nm and PFPE-AA; 160.0 nm, respectively), but the PDI was lower for PFPE-HEMA than PFPE. In detail, the tail of the intensity graph, which has LNPs with Z-average sizes over 1000 nm, emerged from only PFPE slab data. Due to the precipitates in the channel and large particles (microparticles), which cause the loss of products, PFPE-HEMA is suitable for synthesizing LNPs (Figure 3B).

### 3.4. Production of Emulsion Droplets

**Figure 4:**
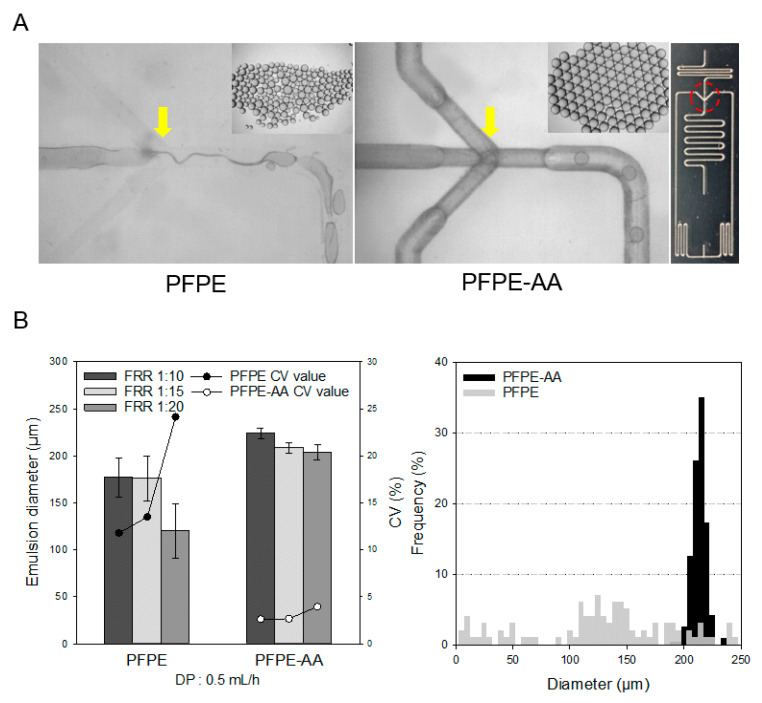
(**A**,**B**) Average diameter and CV value of the emulsions at different flow rates of the continuous phase, which was fabricated by different PFPE-based slabs, and the frequency graph of the emulsion diameter formed at TFR (1.7 mL/h) and FRR (2:15) flow rate conditions.

Figure 4A shows optical microscopy images comparing droplet generation according to PFPE and PFPE-AA. Due to the high refractive index of PFPE-AA, red oil was mixed into DP to observe the channel. In this work, the composition of the continuous and discontinuous phase was (TFR; 1.7 mL/h FRR 2:15). For the microfluidic device using the PFPE slab, DP flowed along the channel wall and generated droplets in random positions. In contrast, uniform droplets were generated at the junction when using the PFPE-AA slab. The results of measuring the average size of the emulsion, flow rate, and average size of the emulsion were in inverse proportion. The CV value was clearly different compared with PFPE (CV value > 40%) and PFPE-AA (CV value < 5%). The frequency graph of emulsion diameter (TFR; 1.7 mL/h FRR 2:15) generated by PFPE shows a broad shape in the line graph, meaning various sizes of emulsion. However, the frequency graph of emulsion diameter using PFPE-AA shows a narrow graph at 200~220 µm diameter, implying the uniform size of the emulsion (Figure 4B). These results confirm that PFPE-AA is appropriate for generating a consistent size of emulsion droplets.

## 4. Conclusions

We developed microfluidic materials with modified hydrophilicity by adding carboxyl additives to PFPE, which has solvent resistance. The produced materials were evaluated by measuring the contact angle, swelling test, and transparency to determine the optimal material for the O/W droplet and fabrication of LNPs. The microfluidic device was manufactured using PFPE-AA and PFPE-HEMA, and LNPs and O/W droplets were produced at a constant production rate with uniform size. The key features of PFPE-AA and PFPE-HEMA are as follows: prevent non-specific adsorption and hydrophobicity recovery, simple fabrication with laser engraving technology for channel engraving, and solvent resistance. Overall, we demonstrated the stable production of LNPs and emulsions without any debris and channel blockage using a PFPE-based microfluidic device with hydrophilic monomers. Improved PFPE is expected to overcome the limitations of conventional PDMS-based microfluidic chips and expand sustainability and productivity in various applications [30,31]. In addition, further research is required into the characteristics of PFPE. For instance, it is not yet known why heat bumps are not generated during laser processing.

## Data Availability

The data are contained within the article.
